# Therapeutic effects of isosteviol sodium on non-alcoholic fatty liver disease by regulating autophagy via Sirt1/AMPK pathway

**DOI:** 10.1038/s41598-022-16119-0

**Published:** 2022-07-27

**Authors:** Ying Mei, Hui Hu, Liangjun Deng, Xiaoou Sun, Wen Tan

**Affiliations:** 1 School of Pharmacy, Jinan University, Guangzhou, 510632 China; 2 YZ Health-Tech Inc., Hengqin District, Zhuhai, 519000 China; 3 Institute of Biomedical and Pharmaceutical Sciences, Guangdong University of Technology, Guangzhou, 510006 China; 4 Jeffrey Cheah School of Medicine and Health Sciences, Monash University Malaysia, 47500 Bandar Sunway, Malaysia

**Keywords:** Biochemistry, Cell biology

## Abstract

Isosteviol sodium (STVNa) is a beyerane diterpene synthesized via acid hydrolysis of stevioside, which can improve glucose and lipid metabolism in animals with diabetes. However, it remains unknown whether STVNa can exhibit a therapeutic effect on nonalcoholic fatty liver disease (NAFLD) and its underlying mechanism. We hypothesize that autophagic initiation may play a key role in mediating the development of NAFLD. Herein, we assessed the effects of STVNa on NAFLD and its underlying mechanisms. The results demonstrated that STVNa treatment effectively ameliorated NAFLD in rats fed high-fat diet (HFD). Moreover, STVNa decreased the expression of inflammation-related genes and maintained a balance of pro-inflammatory cytokines in NAFLD rats. STVNa also reduced lipid accumulation in free fatty acid (FFA)-exposed LO2 cells. In addition, STVNa attenuated hepatic oxidative stress and fibrosis in NAFLD rats. Furthermore, STVNa enhanced autophagy and activated Sirtuin 1/adenosine monophosphate-activated protein kinase (Sirt1/AMPK) pathway both in vivo and in vitro, thus attenuating intracellular lipid accumulation. In summary, STVNa could improve lipid metabolism in NAFLD by initiating autophagy via Sirt1/AMPK pathway. Therefore, STVNa may be an alternative therapeutic agent for treatment of NAFLD.

## Introduction

Western dietary habits and lack of physical activity can increase the risk of various diseases such as diabetes, cardiovascular diseases, renal diseases and cancers^1^. Insulin resistance (IR) can be triggered by a diet containing high-glucose and/or high-fat. Nonalcoholic fatty liver disease (NAFLD) is a closely related to IR, genetic susceptibility, hepatic metabolic stress and liver pathological changes, and the patients have no history of heavy drinking. NAFLD includes a spectrum of hepatocellular damage ranging from hepatic steatosis (HS) to nonalcoholic steatohepatitis (NASH), nonalcoholic liver cirrhosis and hepatocellular carcinoma^2,3^.

Lipid homeostasis is maintained through an interplay between fatty acid metabolism and lipid biosynthesis, and the former is crucial for the progression of HS^4^. Autophagy is a highly conserved cellular degradation process that mediates intracellular homeostasis and improves cell survival^5^. Apart from removing damaged cell organelles and intracellular pathogens, autophagy can regulate lipid metabolism, leading to a decrease in intracellular lipid deposition and prevention of NAFLD^6,7^. Autophagy can be activated by rapamycin or starvation, which in turn breakdowns fatty acid molecules in the mitochondria and subsequently undergoes free fatty acid (FFA) β-oxidation^8^. However, the knockdown of autophagy-related genes is shown to abrogate autophagy and promote hepatic lipid accumulation, leading to the development of HS. Additionally, the initiation of hepatic autophagy may enhance intracellular lipid clearance and reduce susceptibility to NAFLD^9,10^.

Sirtuin 1 (SIRT1) plays an essential role in regulating hepatic lipid metabolism via autophagy induction^11^. The activation of SIRT1-induced autophagy is critical for protecting against HS over dietary restriction, which represents a promising therapeutic target for treating NAFLD^12^. Moreover, pharmacological activation of SIRTI expression can promote lipid droplet biogenesis in autolysosomes and reduce lipid deposition in the liver of obese mice. SIRT1 and adenosine monophosphate-activated protein kinase (AMPK) both regulate each other to exert a synergistic effect. It has been reported that the phosphorylated AMPK (p-AMPK) upregulates SIRT1 expression under stress or energy-depleting conditions, which in turns triggers autophagy to regulate metabolic stress and maintain energy homeostasis^11,12^.

Isosteviol sodium (STVNa) is common sweetener isolated from *Stevia rebaudiana*, which possesses a variety of pharmacological properties, including anti-tumor, anti-inflammatory and neuroprotective activities^13^. Our previous works demonstrated that STVNa not only decreased urinary albumin excretion and glomerular filtration rates, but also improved glucose and lipid metabolism in diabetes^14–16^. However, it remains unclear whether STVNa can exert protective effects on NAFLD. In this study, we investigated the therapeutic effect of STVNa on NAFLD and elucidated its underlying mechanisms by measuring autophagic flux.

## Materials and methods

### Animal and experiments design

Male SD rats (80–100 g, 5–6 weeks old) were supplied by the Animal Research Centre of Guangzhou University of Chinese Medicine (Guangzhou, China). All rats were maintained under standard conditions (24 ± 1 °C and 12-h light/dark cycle). The study is reported in accordance with ARRIVE guidelines. All methods were carried out in accordance with relevant guidelines and all experimental protocols were approved by Committee of Guangzhou University of Chinese Medicine. First, the rats were fed normal laboratory chow for 1 week. Subsequently, they were fed normal diet or high-fat diet (45 kcal% from fat) for 5 weeks. Next, the rats were randomly divided into the following groups (n = 8 per group): normal diet group (ND), HFD group, HFD + STVNa (Dongguan, China; 1, 10, 20 mg/kg two times daily, i.g.) and HFD + Fenofibrate (100 mg/kg/day, i.g), and continuous fed for 5 weeks. Their food intake and body weight (BW) were recorded daily. Oral glucose tolerance test (OGTT) was performed at week 10. After treatment for 12 weeks, the rats were fasted for 12 h and their blood specimens were withdrawn for biochemical analyses. Finally, all rats were killed by CO_2_ inhalation, and their tissues were dissected for real-time quantitative PCR (RT-qPCR) assay and histopathological staining.

### Serum and tissue biochemical analyses

After performing hemodynamic measurements, liver tissue was collected and plasma was obtained by centrifugation. Each liver was rinsed thoroughly with phosphate-buffered saline (PBS) to remove any residual blood. Body weight (BW) and liver weight (LW) were measured, and the ratio of LW:BW was calculated. Subsequently, the liver samples were sectioned at 2 cm thickness, and then snap-frozen in liquid nitrogen for subsequent analyses. Meanwhile, the plasma specimens were kept at − 80 °C until use. The levels of total cholesterol (TC), triglyceride (TG), high-density lipoprotein-cholesterol (HDL-C), low density lipoprotein-cholesterol (LDL-C), aspartic acid transaminase (AST), and alanine aminotransferase (ALT) in serum were measured using the commercially available Kit-WST (Nanjing Jiancheng, China). After overnight fasting, the rats were subjected to orally glucose tolerance test (OGTT) on week 10. Tail vein blood was obtained, and fasting blood glucose level was detected using an Accu-Chek glucometer (Roche Diagnostics, Mannheim, Germany).

### ELISA measurements

The levels of insulin, CXCL10, CK18-M30 and leptin were detected with ELISA kits (Nanjing Jiancheng, China) by following the kit’s protocols. All experiments were performed in triplicate.

### Measurements of malondialdehyde (MDA)/glutathione (GSH) and superoxide dismutase (SOD) activities in liver

To determine Liver levels of GSH, SOD, and MDA, the supernatant of the homogenized Liver tissues for analysis was collected by centrifugation at 4000 rpm for 10 min at 4 °C. The concentration of proteins was quantified with the bicinchoninic acid (BCA) kit (Ding Guo, Beijing, China). MDA, GSH and SOD activities were detected using MDA, GSH Assay Kit-WST and SOD Assay Kit-WST (Nanjing Jiancheng, China) according to the manufacturer’s protocols.

### OGTT measurement

After 6 h of fasting, blood glucose in the tail vein was determined using the Accu-check glucometer. The blood samples were taken at 0, 15, 30, 60, 90, and 120 min after oral administration of 2 g/kg D-glucose.

### RT-qPCR assays

RNeasy lipid tissue mini kit (Qiagen, Germany) was utilized to extract total RNA from liver tissues. The purity and yield of RNA samples were evaluated using a NanoDrop 2000/2000c spectrophotometer (Thermo Scientific, USA). cDNA synthesis was conducted with RT2 strand kit (Qiagen, Germany). The expression levels of inflammation- and fibrosis-related genes were detected with SYBR Green qPCR Mastermix (Qiagen, Germany) using a Custom RT2 profiler PCR array. RT-qPCR was conducted on a Biosystem StepOne Plus thermal cycler (Applied Biosystems, USA). The fold-change in the mRNA expression of each target gene was calculated based on the 2^ΔΔCt^ method. The primer sequences (Table 1) were synthesized by Generay Biotech.

**Table 1:** Primer sequences employed in RT-qPCR assays.

Gene	Forward (5′ → 3′)	Reverse (5′ → 3′)
Collgen I	GCCAAGAAGACATCCCTGAAGT	ACATCAGGTTTCCACGTCTCACCA
Collgen III	TCCTGGTGGTCCTGGTACTG	AGGAGAACCACTGTTGCCTG
TGFβ	TACCATGCCAACTTCTGTCTGGGA	TGTGTTGGTTGTAGAGGGCAAGGA
TNFα	ATGGCCTCCCTCTCATCAGT	CTTGGTGGTTTGCTACGACG
IL6	TTCCATCCAGTTGCCTTCTTG	TTGGGAGTGGTGTATCCTCTGTGA
IL1β	CTTCCCCAGGGCATGTTAAG	ACCCTGAGCGACCTGTCTTG
GADPH	AGCCAAAAGGGTCATCATCT	GGGGCCATCCACAGTCTTCT

### Immunohistochemical assays

Immunohistochemical (IHC) analysis of α-SMA was performed with mouse monoclonal antibody against α-SMA. Following deparaffinization and rehydration, 0.01 M boiled citrate buffer (pH 6.0) was added into each section, and heated at 97 ± 1 °C for 10 min to achieve antigen retrieval. The sections were cooled to room temperature (RT), followed by incubation with 3% H_2_O_2_ (1% in 0.01 M PBS, v/v) for 10 min to remove endogenous peroxidase. After placing in PBS and blocking with 5% albumin bovine serum (Sigma-Aldrich, MO, USA) at 37 °C for 30 min, the sections were exposed to the primary antibody mouse anti-α-SMA (1:3000; Abcam, Cambridge, UK) at 4 °C overnight. Subsequently, the sections were pre-warmed to RT for 30 min and incubated at 37 °C for 30 min. After rinsing in PBS, the sections were exposed to the secondary antibody Envision System-HRP-conjugated anti-mouse IgG (DAKO, Denmark) at 37 °C for 40 min. After the final wash, the sections were stained with 3,3′-diaminobenzidine (DAB) and recorded using a DAB-enhanced liquid substrate system (DAKO). Lastly, the sections were counterstained with hematoxylin. To verify the specificity of IHC assay, the primary antibody was replaced by Mouse (G3A1) mAb IgG1 Isotype Control (Cell Signaling Technology, MA, USA) as a negative control. The densitometry analysis of positive staining was conducted with Image-Pro Plus tool.

### Cell culture and treatment

Human LO2 cell line were purchased from china Type Culture Collection (Chinese Academy of Sciences, Shanghai, China), cultured with high-glucose Dulbecco’s Modified Eagle Medium (DMEM) containing streptomycin (100 U/mL), penicillin (100 U/mL) and calf serum (10%, v/v). To prevent differentiation, the cells were grown under subconfluent conditions. Differentiation medium [DMEM/high-glucose containing 5 μg/mL insulin, 10% fetal bovine serum (FBS), 5 mM MIX and 0.4 μg/mL dexamethasone] was then added to induce cell differentiation. On the next day, the cells was incubated with the differentiation medium without 5 mM MIX and 0.4 μg/mL dexamethasone for 2 days, and then replaced by DMEM/high-glucose containing only 10% FBS for another 4–6 days. The differentiated cells were verified by lipid droplet quantification through Oil Red O (ORO) staining.

### Cell viability assay

Cell viability was assessed using the Cell Counting Kit-8 (CCK8) assay (Beyotime Co., Ltd., Shanghai, China). First, LO2 cells were seeded in a 96-well plate at 37 °C and 5% CO_2_. When the cell fusion reached 80%, LO2 cells were stimulated with 1 mM FFA (OA:PA = 2:1) for 24 h, then with different concentrations of STVNa for 24 h; Then, the CCK-8 diluent (0.5 mg/mL) was added at a volume of 100 μL per well. Lastly, the optical density was measured at 450 nm after incubation for 1–4 h at 37 °C.

### Measurement of intracellular ROS

The levels of ROS were determined by DCFH-DA (Sigma-Aldrich, USA), a non-fluorescent, cell-permeable probe that is de-esterified and then oxidized to 2′,7′-dichlorofluorescein with high fluorescence. The LO2 cells (70–80% confluence) were treated accordingly, and the accumulation of intracellular ROS was evaluated with DCFH-DA. Briefly, the cells were incubated with 5 μmol/L DCFH-DA at 37 °C for 30 min. After washing 3 times with PBS, the fluorescence intensities were examined using a confocal microscope (Carl Zeiss, Germany). The image analyses performed to quantify intracellular ROS were used software Image J (https://imagej.en.softonic.com/).

### Evaluation of mitochondrial membrane potential (Δψ)

JC-1 (Sigma-Aldrich, Germany) is a cationic dye that shows potential-dependent shift in fluorescence emission from 525 nm (green) to 590 nm (red). A decrease in red:green fluorescence ratio indicates the depolarized mitochondria. The LO2 cells in different treatment groups were incubated with 10 μg/mL JC-1 staining solution at 37 °C for 15 min. After washing 3 times with PBS, the cells were observed under the confocal microscope. Δψ denotes the ratio of red:green fluorescence intensities. The red and green fluorescence intensity of images was analyzed by software Image J (https://imagej.en.softonic.com/).

### ORO staining

The differentiated cells were exposed to 1, 5 and 10 μM of STVNa or DMSO (1:1000) for 8 days. The accumulation of lipids was assessed by ORO staining. After fixing in 10% formalin for 1 h, the cells were stained with ORO solution (3 parts of 0.5% ORO dye in isopropanol to 2 parts of water) for 1 h. To remove unbound dye, the cells were rinsed 3 times with 60% isopropanol, and then examined at 50 × magnification using an Axiovert 40 CFL microscope (Carl Zeiss). Finally, ORO-stained intracellular lipids were extracted with isopropanol, and the optical density was measured at 492 nm.

### Western blot analysis

RIPA lysis buffer (Beijing Dingguo Technology) containing phosphatase and protease inhibitors was used to extract total protein the differentiated cells after 8 days of treatment. The protein concentrations were quantified using a BCA kit (Beijing Dingguo Technology). Forty micrograms of the extracted proteins were subjected to SDS-PAGE (110 V, 2 h), followed by transferring (110 V, 90 min) onto polyvinylidene difluoride membranes (Millipore, USA). After blocking with 5% skimmed milk at RT for 1 h, the membranes were incubated with primary antibodies as follows: AMPK (rabbit polyclonal, 1:1000, #sc-61, Santa Cruz), P-AMPK (rabbit polyclonal, 1:1000, #sc-61, Santa Cruz), microtubule-associated protein 3 (LC3 rabbit polyclonal, 1:1000, #sc-61, Santa Cruz), P62 (rabbit polyclonal, 1:1000, #sc-61, Santa Cruz), Sirt1 (mouse monoclonal, 1:500, #sc-7273, Santa Cruz) and Sirt3 (rabbit polyclonal, 1:1000, #sc-61, Santa Cruz) at 4 °C overnight. After rinsing with TBST buffer, the membranes were incubated with the corresponding HRP-conjugated secondary antibodies (DAKO, Denmark) at RT for 45 min. After washing 3 times with TBST buffer, the protein blots were visualized with ECL kit (Beijing Dingguo Technology). Next, the membranes were detected using an enhanced chemiluminescence detection system (Pierce, Rockford, USA).

### Autophagic flux assay

The LO2 cells were infected with red-green fluorescent proteins (RFP-GFP)-LC3 adenovirus (Hanbio, Shanghai, China), and then incubated with/without STVNa (100 µg/mL) and FFA (1 mM). After the autophagosome–lysosome fusion, the RFP signals remain stable, while the GFP signals are quenched within the lysosomes. The overlapping between RFP-LC3 and GFP-LC3 results in an increase in yellow puncta (autophagosomes) and a decrease in red puncta (autolysosomes). The fluorescence intensities were examined at 550–590 nm (RFP-LC3) and 395–495 nm (GFP-LC3) using the confocal laser scanning microscope.

### Statistical analysis

All values were shown as means ± standard error of the mean (SEM). One-way ANOVA followed by Bonferroni’s multiple comparison tests were performed with GraphPad Prism v5.0 (https://www.graphpad.com/). Level of statistical significance was set at *P* < 0.05.

## Results

### HFD induces the development of NAFLD in rats

**Figure 1:**
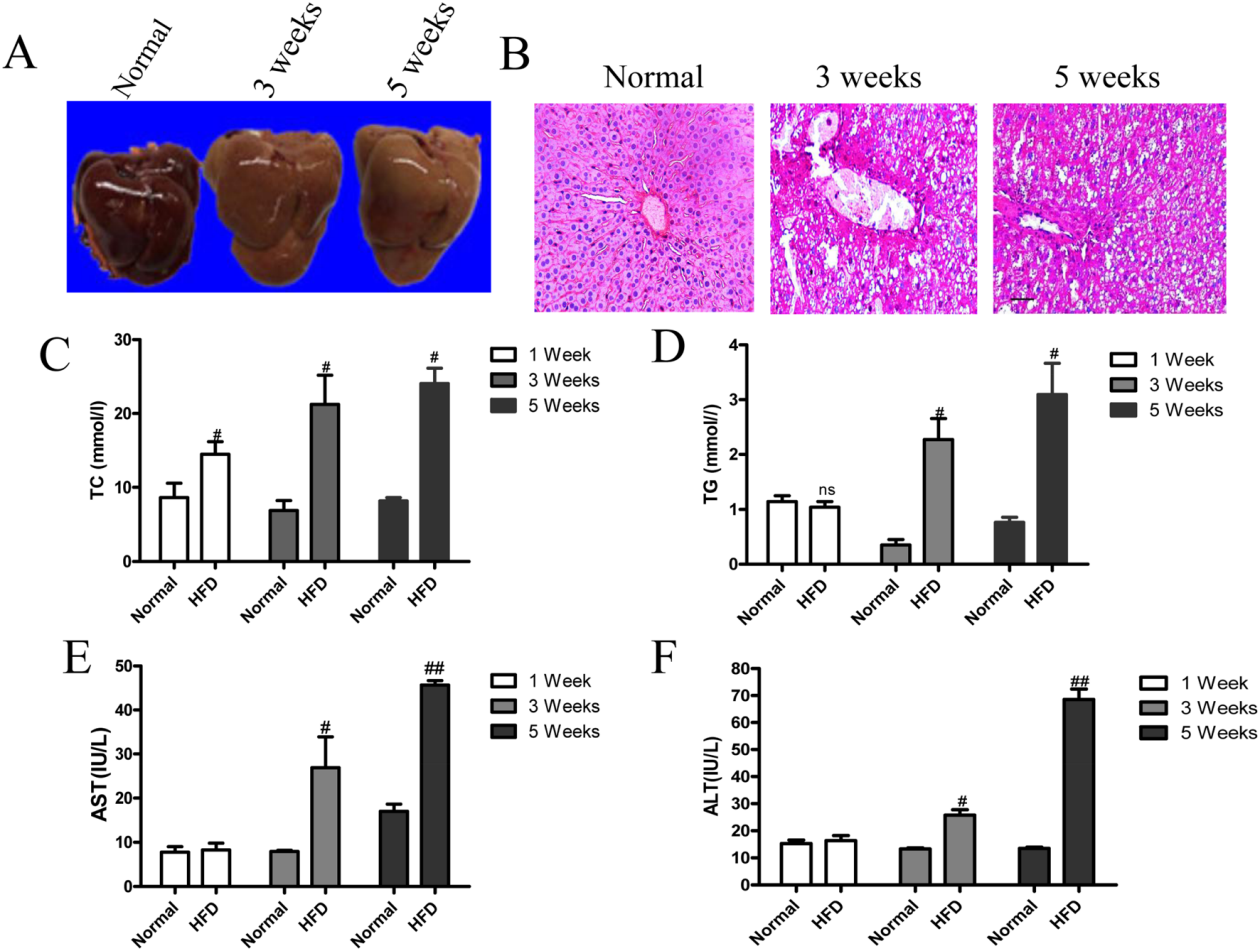
Progression of NAFLD is induced by HFD. (**A**) The characterization of liver morphology. (**B**) H&E staining revealed the development of NAFLD induced by HFD. Scale bar in A = 50 µm. (**C**–**F**) The levels of TC, TG, ALT and AST of HFD-fed rats at weeks 1, 3 and 5. Mean ± SEM. ^#^*P* < 0.05, ^##^*P* < 0.01 vs. ND-fed rats.

To clarify the dynamical process of NAFLD in rats, the morphological characteristics of HFD-fed rats were recorded at various time points following induction of NAFLD. The results showed that, with increasing time, the normal dark red color gradually faded to light red, and the liver edge gradually passivated from smooth (Fig. 1A). Moreover, the rats exhibited liver dysfunction at 5 weeks after HFD treatment when compared to the ND-fed rats. Very small vacuoles were found in the cytoplasm and inflammatory cells, and were infiltrated in portal areas and hepatic lobules by H&E staining (Fig. 1B). Meanwhile, HFD significantly increased the hepatic levels of TG and TC after HFD treatment at weeks 1, 3 and 5 (Fig. 1C,D). We also observed that the levels of AST and ALT were higher in HFD-fed rats than in ND-fed rats, indicating the damaged liver function after NAFLD (Fig. 1E,F).

### STVNa alleviates NAFLD in HFD-fed rats

**Figure 2:**
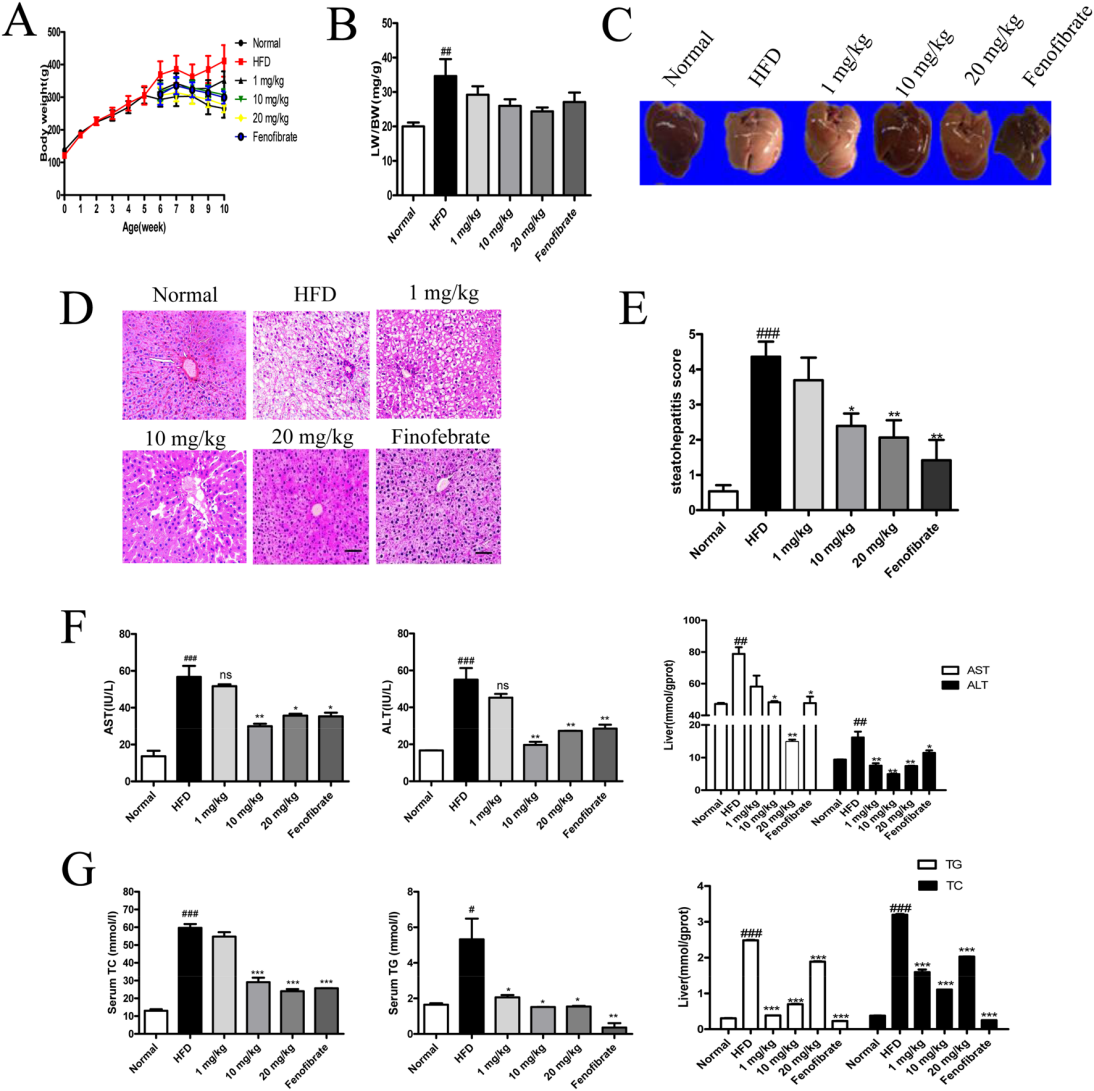
STVNa alleviates NAFLD in HFD-fed rats. (**A**) The animals fed a HFD for 10 weeks and exposed to 1, 10 or 20 mg/kg/day STVNa for 5 weeks. (**B**) BW was recorded every week, and BW gain was determined at week 10. STVNa could result in a weight loss in HFD-fed rats (*P* > 0.05). (**C**) The characterization of liver morphology at week 10. (**D**) H&E staining revealed NAFLD and inflammatory cell infiltration (black arrow). Scale bar = 50 µm. (**E**) NAFLD activity scores (lobular inflammation and hepatic steatosis). (**F**,**G**) The levels of AST, ALT, TC and TG of HFD-fed rats treated with/without STVNa. Mean ± SEM (n = 6). *P < 0.05, **P < 0.01, ***P < 0.001 vs. HFD-fed rats; ^#^*P* < 0.05, ^##^*P* < 0.01 and ^###^*P* < 0.01 vs. ND-fed rats.

High-fat Western diet is commonly used to establish an obesity-related NAFLD animal model^17^. STVNa attenuated abnormal BW gain and decreased the ratio of LW:BW in HFD-fed rats, indicating that STVNa could be used to treat NAFLD (Fig. 2A,B). The HFD-fed rats showed a remarkable increase in liver TG compared to the ND-fed rats, which could be improved by STVNa treatment (Fig. 2G). With respect to the changes in lipid metabolism, the high contents of TC and TG were decreased by STVNa in HFD-fed rats (Fig. 2G). Similarly, the high levels of AST and ALT were attenuated by STVNa treatment (Fig. 2F). In addition, STVNa treatment obviously decreased the areas of NAFLD as well as the numbers of infiltrating inflammatory cell clusters and ballooning hepatocytes, thus partially reversing the morphological alterations in the liver tissues of NAFLD rats (Fig. 2C–E).

### Effect of STVNa on IR in NAFLD rats

**Figure 3:**
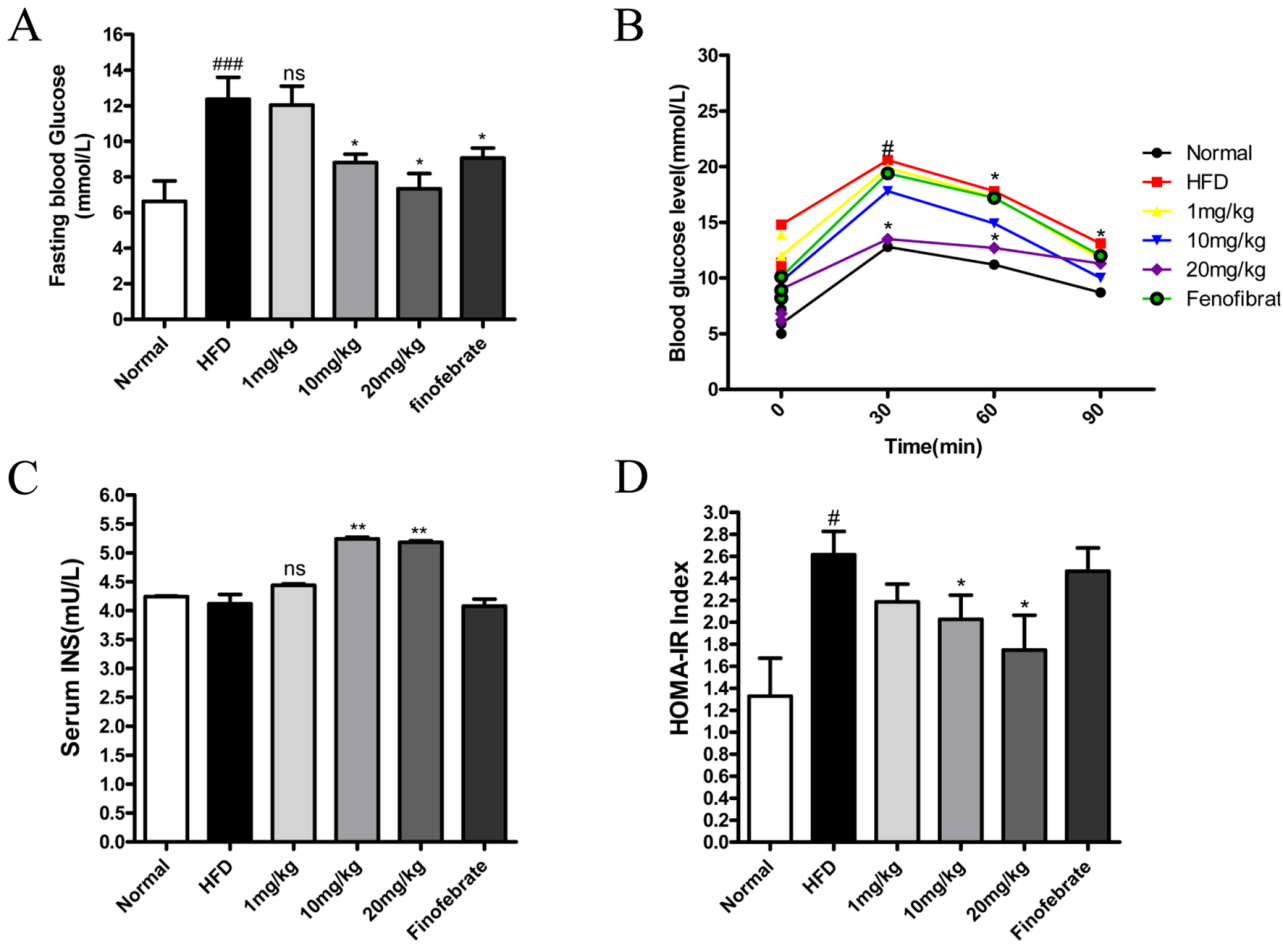
Effect of STVNa on IR in HFD-induced NAFLD. (**A**) The content of fasting blood glucose. (**B**) IPGTT was conducted to determine the effect of STVNa on glucose metabolism. (**C**) Serum of insulin content. (**D**) HOMA-IR indices. After 5 weeks of STVNa intervention, the HFD-fed rats had an enhancement in HOMA-IR scores (*P* < 0.05). Mean ± SEM. *P < 0.05, **P < 0.01 vs. HFD-fed rats; ^##^*P* < 0.01, ^###^*P* < 0.001 vs. ND-fed rats.

IR is a major risk factor of NAFLD that is caused by excess lipid deposition^18^. Therefore, OGTT was conducted after treated with 2 g/g BW i.p. glucose. In addition to fenofibrate, STVNa remarkably enhanced glucose tolerance via the IPGTT. Moreover, the area under the curve revealed that STVNa markedly improved glucose tolerance in HFD-fed rats (Fig. 3B). NAFLD rats had increased and decreased levels of blood glucose (Fig. 3A) and insulin (Fig. 3C), respectively, after 10 weeks of HFD treatment. Notably, STVNa reduced blood glucose and elevate insulin levels in HFD-fed rats. Furthermore, we calculated the IR index through the formula, and the results showed that the model group had IR, while the IR in the administration group was improved to a certain extent (Fig. 3D). Collectively, these findings demonstrate that STVNa effectively improves NAFLD-related symptoms in HFD-fed rats (Supplementary Information).

### Effect of STVNa on pro-inflammatory cytokine secretion in NAFLD

**Figure 4:**
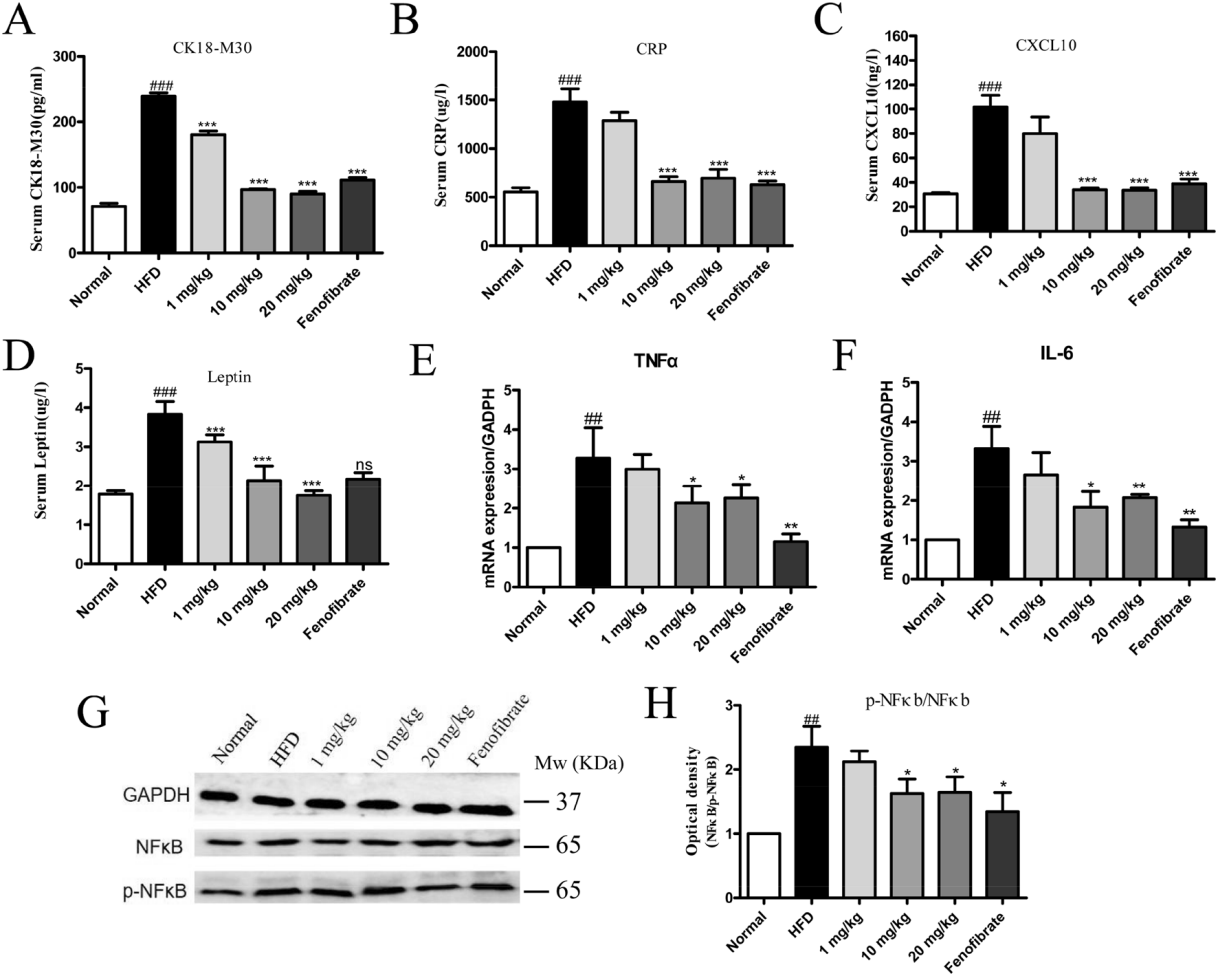
STVNa maintains a balance of pro-inflammatory cytokine levels in NAFLD in rats. (**A**–**D**) The levels of CK18-M30, CRP, CXCL10 and Leptin. (**E**,**F**) Quantitative analysis of TNFα and IL-6 levels. The mRNA levels of TNFα and IL-6 detected by RT-qPCR. (**G**) The protein levels of NFκb and p-NFκb examined by Western blotting (n = 3). (**H**) The levels of p-NFκb/NFκb after STVNa treatment. Mean ± SEM. *P < 0.05 vs. HFD-fed rats; ^#^*P* < 0.05 vs. ND-fed rats.

Studies have found that one of the main characteristics of NAFLD is inflammatory response. Therefore, we analyzed the levels of CRP (c-reactive protein), a marker of acute inflammatory response, CXCL10, CK-18M30 and Leptin in rats via ELISA. Notably, the levels of CRP and CXCL10 were remarkably higher (*P* < 0.05) in HFD-fed rats than in ND-fed rats (Fig. 4). After treatment with STVNa or fenofibrate for 5 weeks, the levels of CRP and CXCL10 were markedly decreased when compared to HFD-fed rats (*P* < 0.05). Additionally, TNF-α and IL-6 play a crucial role in mediating inflammatory responses. Hence, the expression levels of TNF-α and IL-6 genes through RT-qPCR assay. The findings demonstrated STVNa could downregulate the expression levels of inflammation-related genes. It is well accepted that NF-κB is the most classic inflammatory signaling pathway, and the protein P-NF-κB is highly expressed in response to inflammation and oxidative stress. In response to environmental stimuli, the NF-κB pathway is activated, and some of its components enter the nucleus, leading to a series of inflammatory reactions that damage cells. Therefore, we used Dingguo biological protein extraction kit to assess the corresponding total protein level, and determined the protein level of NF-κB in liver tissue by Western blotting. The data indicated that the protein level of P-NF-κB was remarkably higher in HFD-fed rats than in ND-fed rats (*P* < 0.05), and NF-κB was significantly reduced after STVNa treatment (*P* < 0.05), as shown in Fig. 4G,H. Taken together, our findings demonstrated that HFD-induced NAFLD increased the levels of pro-inflammatory cytokines, and STVNa could reduce these levels and maintain a balance of pro-inflammatory cytokines in NAFLD rats.

### Effect of STVNa on lipid overaccumulation in NAFLD

**Figure 5:**
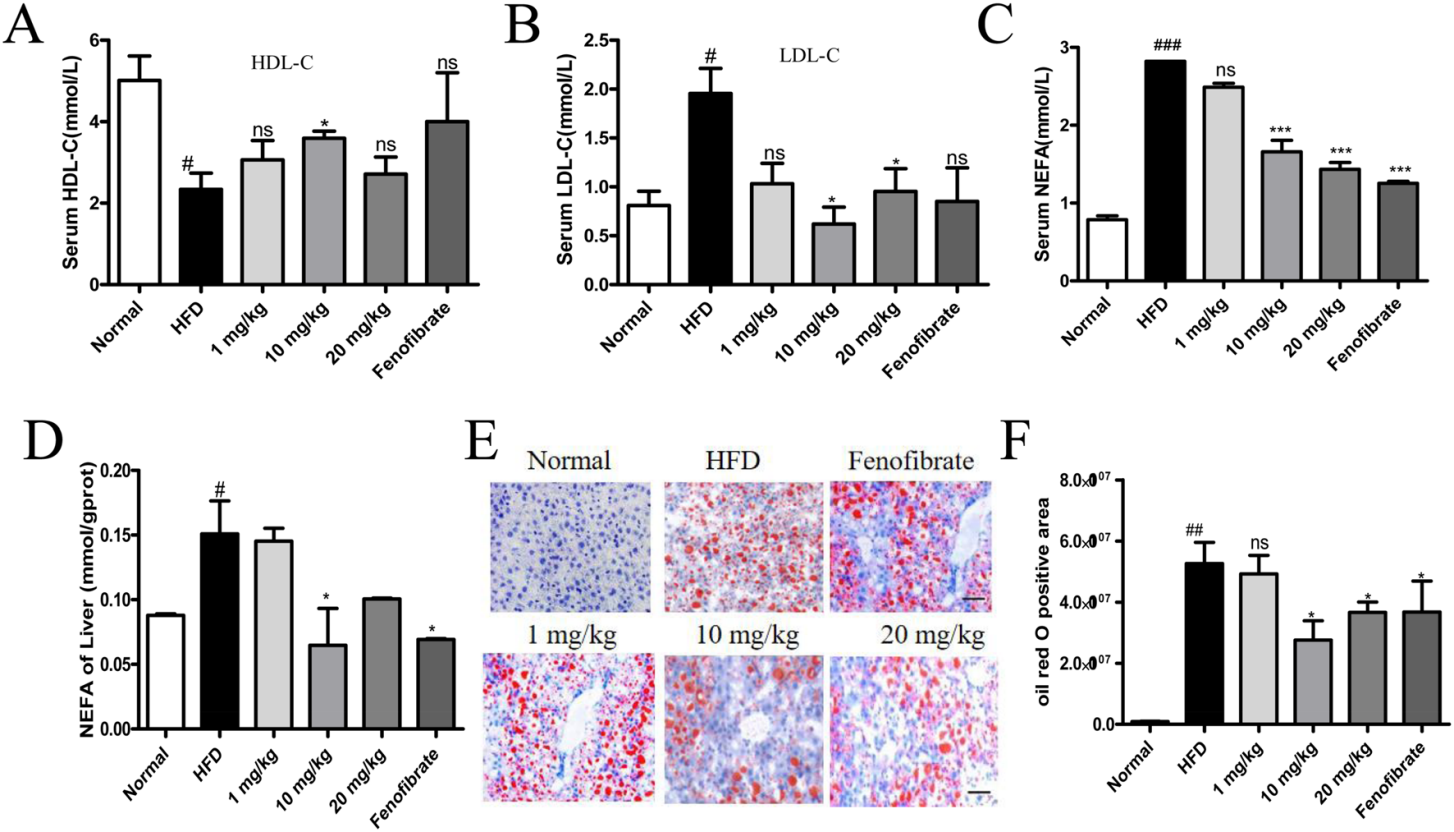
STVNa improves lipid metabolism in NAFLD rats. (**A**,**B**) The levels of HDL-C and LDL-C. (**C**,**D**) The levels of NEFA in blood and liver tissue. (**E**) ORO-stained sections HFD-fed rats treated with/without STVNa. Scale bar = 50 µm. (**F**) Positive ORO-stained areas. Mean ± SEM. *P < 0.05 vs. HFD-fed rats; ^#^*P* < 0.05 vs. ND-fed rats.

Compared with the ND-fed rats, the levels of LDL-C and HDL-C were remarkably increased and decreased (*P* < 0.05), respectively, in HFD-fed rats (Fig. 5A,B). Treatment with STVNa and fenofibrate markedly attenuated the deposition of lipids in HFD-fed rats (*P* < 0.05). Meanwhile, we also detected the content of free fatty acids (NEFA) in the blood and tissue samples of HFD-fed rats. Notably, the concentrations of NEFA were obviously lower in STVNa group than in HFD group (Fig. 5C,D). Furthermore, ORO staining also revealed that HFD expanded the ORO-positive areas, which could be suppressed by STVNa treatment in a concentration-dependent fashion (Fig. 5E,F). These findings suggest that the long-term consumption of HFD diet can induce the development of dyslipidemia in NAFLD rats, and STVNa treatment alleviates these symptoms. Taken altogether, STVNa can reduce fat accumulation in liver cells, promote fat metabolism and reduce lipid toxicity.

### STVNa attenuates hepatic oxidative stress and fibrosis

**Figure 6:**
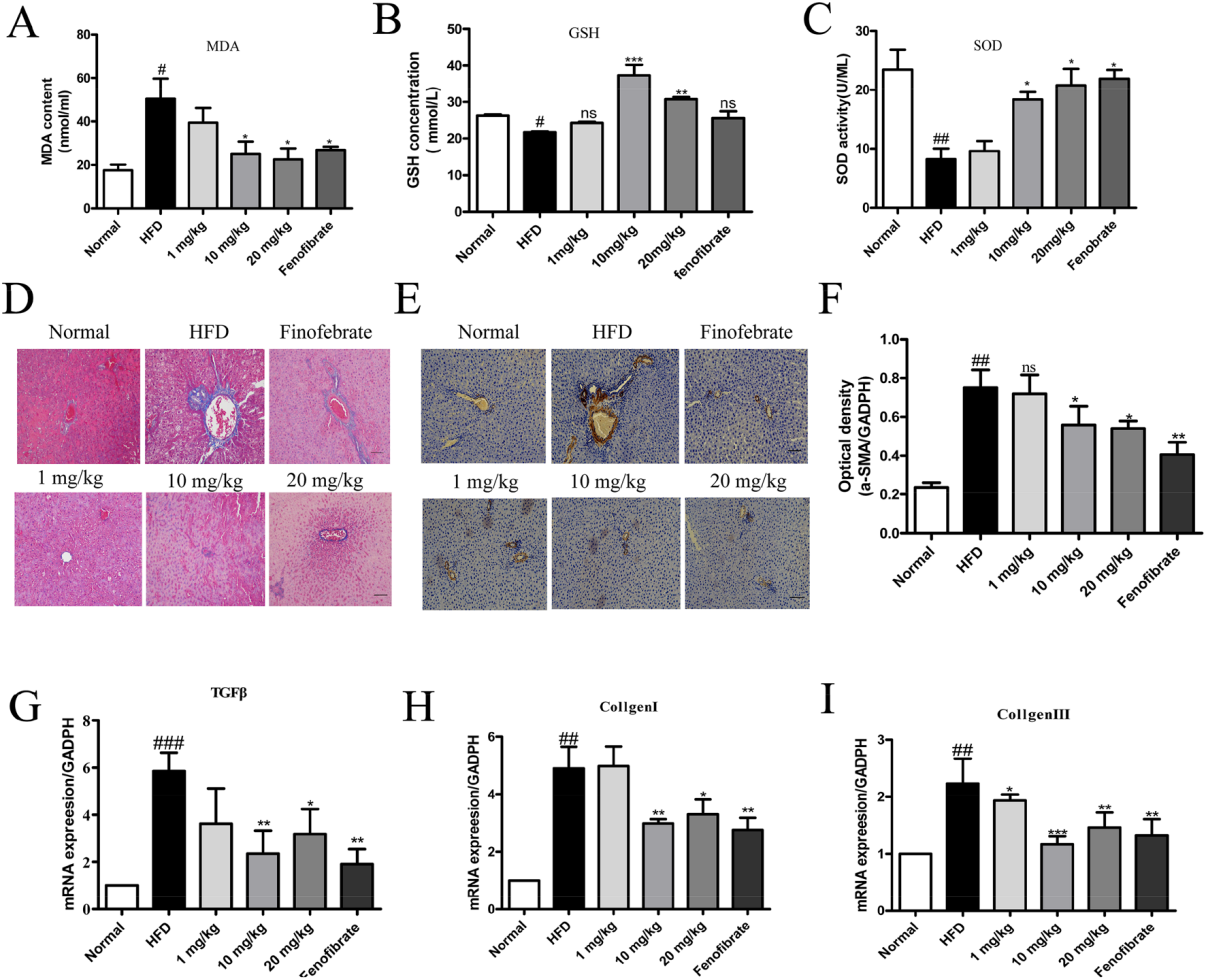
STVNa suppresses hepatic oxidative stress and fibrosis. (**A**–**C**) Total MDA content, GSH level and GSH level in NAFLD rats at week 10. (**D**) Masson staining. (**E**,**F**) IHC measurements of α-SMA. 200 × magnification; Scale bar = 100 µm. (**G**–**I**) The expression levels of TGFβ, Collagen I and Collagen III in NAFLD rats after 5 weeks of STVNa treatment (n = 3 per group). *P < 0.05, **P < 0.01 and ***P < 0.001 vs. HFD-fed rats; ^##^*P* < 0.01 and ^###^*P* < 0.001 vs. ND-fed rats.

Total glutathione (GSH) was apparently declined in the liver tissues of NAFLD rats at week 10, which could be restored by STVNa treatment (Fig. 6A,B). The level of SOD exhibited a similar trend (Fig. 6C). Therefore, total MDA level in liver tissues was markedly higher in HFD-fed rats than in ND-fed rats, while that in STVNa or fenofibrate treatment group was significantly lower. All these oxidative stress-related biomarkers were obviously downregulated by STVNa treatment. A significant decrease in liver collagen accumulation was observed in HFD-fed rats compared with ND-fed rats. Moreover, collagenous fiber formation, fragmented hepatic nuclei and inflammatory cell infiltration were detected in HFD-fed rats by Masson staining (Fig. 6D). The HFD-induced damage was markedly reversed by STVNa treatment. In addition, IHC analysis revealed that α-SMA, another marker of fibrosis, was remarkably elevated in the liver tissues of NAFLD rats at 10 weeks (Fig. 6E,F). Consistent with the pathological results, the mRNA levels of Collagen I, Collagen III and TGFβ were markedly upregulated in the liver tissues of HFD-fed rats, but returned to baseline levels after treatment with STVNa (Fig. 6G–I).

### STVNa enhances autophagy and activates Sirt1/AMPK pathway in HFD-fed rats

**Figure 7:**
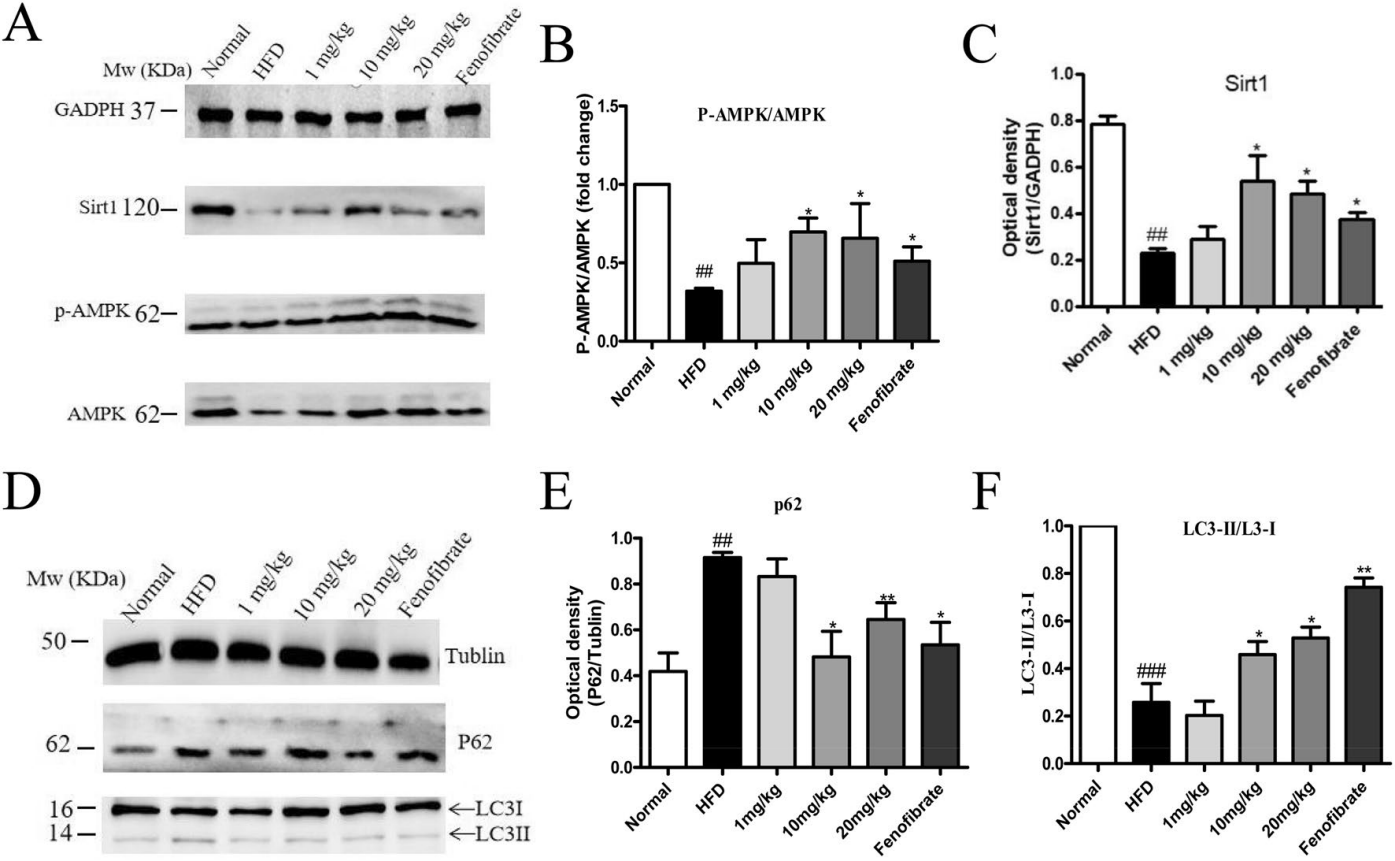
STVNa enhances autophagy and activates Sirt1/AMPK pathway in HFD-fed rats. (**A**–**C**) Western blotting and quantification of SIRT1, AMPK and p-AMPK expression in HFD-fed rats treated with/without STVNa. Mean ± SEM (*n* = 6). (**D**–**F**) Western blotting and quantification of p62, LC3B-II and ratio of LC3B-II:LC3B-I in HFD-fed rats with/without STVNa. Mean ± SEM (*n* = 6). *P < 0.05, **P < 0.01 vs. HFD-fed rats; ^##^*P* < 0.01, ^###^*P* < 0.001 vs. ND-fed rats.

It has been reported that prolonged exposure to an HFD challenge can suppress the positive effects of SIRT1 and AMPK on autophagy in liver cells, leading to excessive lipid deposition and reduced autophagy activity. Western blot analysis was conducted to examine the protein levels of autophagy-related mediators in the liver tissues (Fig. 7A). The densitometry analysis revealed that the expression of SIRT1, a downstream target of AMPK, and the ratio of p-AMPK:AMPK (Fig. 7B,C), were markedly reduced in HFD-fed rats compared with ND-fed rats, indicating that the regulation of autophagy was suppressed by hepatic lipid accumulation.

Similarly, the level of LC3B-II and ratio of LC3B-II:LC3B-I was reduced, while p62 level was remarkably elevated in HFD-fed rats compared to ND-fed rats (Fig. 7D). After treatment with STVNa, SIRT1 expression and p-AMPK:AMPK ratio were reversed in NAFLD rats. Moreover, STVNa treatment markedly increased LC3B-II level and LC3B-II:LC3B-I ratio, while reduced p62 level in HFD-fed rats (Fig. 7E,F). The findings imply that prolonged treatment with STVNa can induce hepatic autophagy via Sirt1/AMPK pathway.

### STVNa reduces FFA-induced lipid deposition in LO2 cells

**Figure 8:**
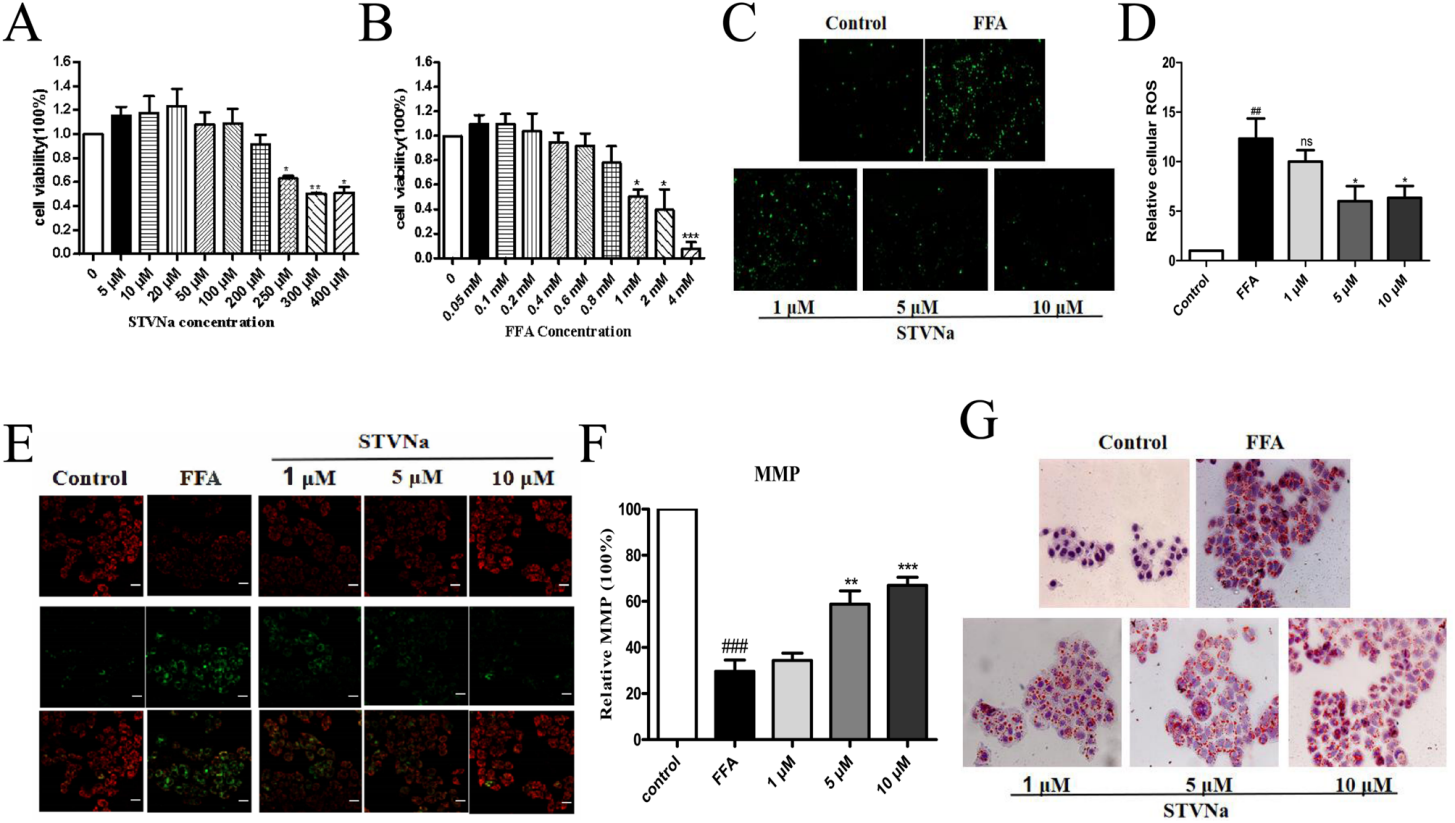
STVNa reduces lipid accumulation in LO2 cells after FFA exposure. (**A**,**B**) The viability of LO2 cells assessed by CCK-8 assay. (**C**) Fluorescence measurement of ROS levels. Scale bar = 100 μm. (**D**) Graphs showing the changes in ROS levels. (**E**) Representative images of three independent experiments. Fluorescence staining of mitochondrial membrane potential by JC-1 dye. Scale bar = 50 μm. (**F**) Graphs showing the red-to-green (R/G) fluorescence intensities. (**G**) Lipid accumulation in LO2 cells determining by ORO staining. Mean ± SEM. *P < 0.05, **P < 0.01, ***P < 0.001 vs. FFA-exposed cells; ^##^*P* < 0.01, ^###^*P* < 0.001 vs. control cells.

The results of CCK-8 assay showed that the viability of LO2 cells was markedly reduced compared with control cells after 48 h of FFA exposure (*P* < 0.05). However, STVNa ameliorated FFA-induced cell viability at 1, 5, 10 μmol/L (Fig. 8A,B). Hence, these concentrations were selected for subsequent experiments. To assess whether STVNa can decrease FFA-induced ROS accumulation, the levels of ROS were determined. The results demonstrated that the levels of ROS were remarkably higher in FFA-exposed LO2 cells than in control cells, suggesting that FFA could induce ROS production. Interestingly, STVNa markedly attenuated the levels of ROS after exposure for 48 h (Fig. 8C,D), implying that STVNa treatment could reduce FFA-induced ROS accumulation.

Mitochondrial dysfunction is indicated by a reduction in Δψ. The effect of STVNa on Δψ was assessed by JC-1 dye. The mitochondrial membrane potential of FFA-exposed cells was noticeably decreased compared to that of control cells (Fig. 8E,F). Interestingly, treatment with 1, 5 or 10 μmol/L STVNa markedly enhanced Δψ a concentration-dependent fashion (*P* < 0.05).

Furthermore, the lipid clearance effects of STVNa on LO2 cells were evaluated. Exposure to 1 mM FFA for 24 h remarkably promoted lipid accumulation. However, pretreatment with 0.5 mM STVNa for 2 h markedly decreased FFA-induced lipid accumulation in LO2 cells, as revealed by ORO staining (Fig. 8G). Besides, 1 mM FFA significantly induced lipid accumulation in LO2 cells after exposure for 8 h (*P* < 0.001). However, pretreatment with 0.5 mM STVNa markedly reduced FFA-induced lipid accumulation.

### STVNa alleviates NAFLD by reinforcing autophagy in FFA-exposed LO2 cells

**Figure 9:**
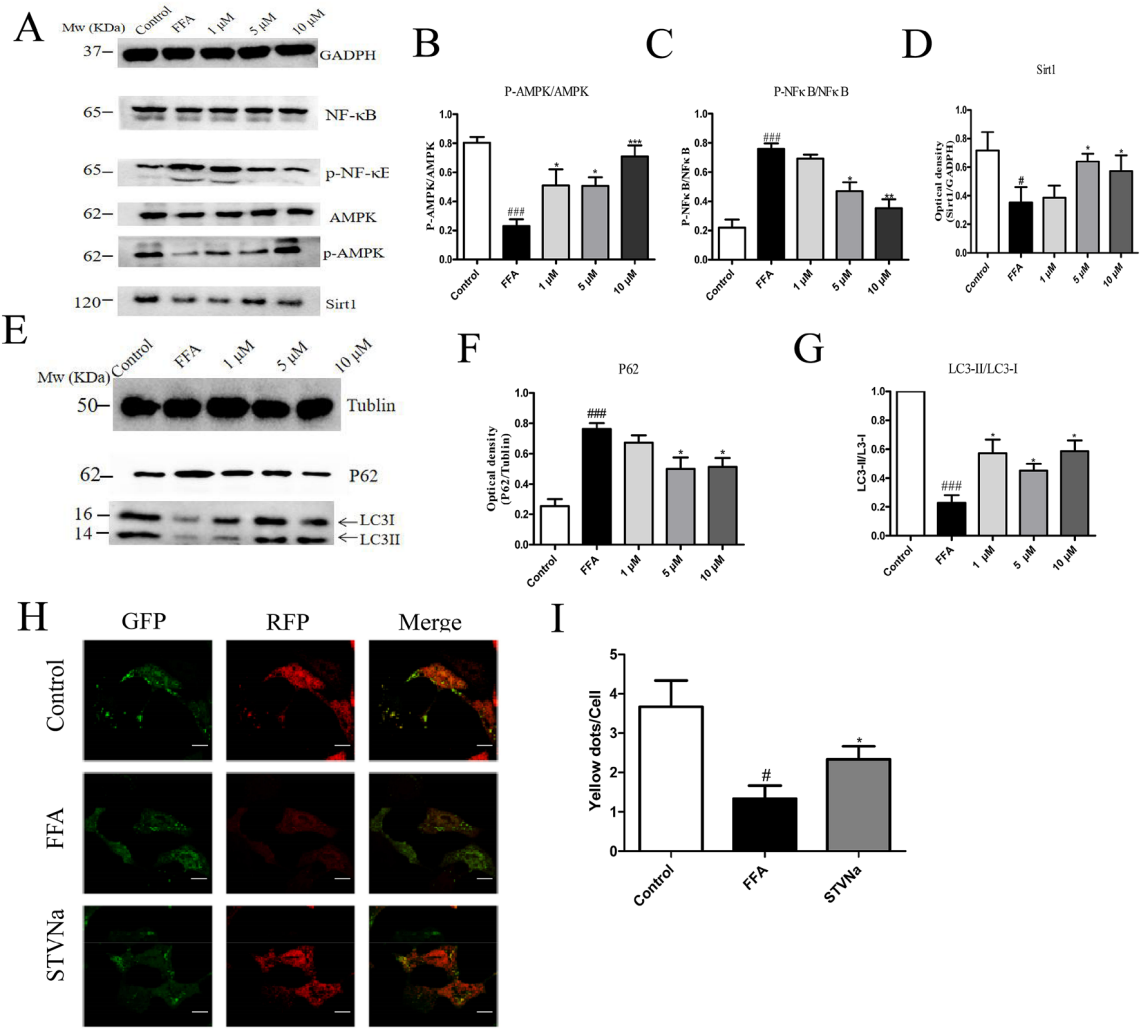
STVNa alleviates NAFLD by reinforcing autophagy in FFA-exposed LO2 cells. (**A**–**D**) Western blotting and quantification of SIRT1, AMPK and p-AMPK expression in FFA-exposed LO2 cells after exposure to STVNa for 24 h (n = 3 per group). (**E**–**G**) Western blotting and quantification of P62 and LC3B-II expression in FFA-exposed LO2 cells. (**H**) Autolysosomes (red puncta) and autophagosomes (yellow puncta) in FFA-exposed LO2 cells after exposure to STVNa for 24 h. Scale bar = 2.5 µm. (**I**) The numbers of autolysosomes and autophagosomes in each LO2 cell were counted. Mean ± SEM. **P < 0.01 vs. FFA-exposed cells; ^#^*P* < 0.05, ^##^*P* < 0.01 vs. control cells.

To assess whether hepatic autophagy is the mechanism corresponded for the protective effect of STVNa on NAFLD, the protein levels of P62, LC3-II/LC3-I, AMPK, and p-AMPK were detected in STVNa treatment (Fig. 9A,C) and FFA exposure (Fig. 9B,D) groups both in vitro and in vivo. It was observed that the levels of P62, LC3-II, AMPK, and p-AMPKα1 were downregulated in NAFLD cells and HFD-fed rats mice (Fig. 9A,B) when compared to those in control groups (Fig. 8C,D). In addition, treatment with STVNa elevated the levels of p62, LC3-II/LC3-I, AMPK and p-AMPK, while reduced Sirt1 expression (Fig. 9E–G).

In this study, we first used RFP-GFP-LC3-transfected LO2 cells to verify the effect of STVNa on autophagy. Autophagy is a dynamic cellular degradation process, which involves the fusion of autophagosomes with lysosomes to produce autolysosomes, and subsequent degradation of the cytoplasmic components by lipases and proteases within the autolysosomes. The autophagic flux analysis revealed that STVNa induced the formation of autolysosome (red puncta) and autophagosome (yellow puncta) in RFP-GFP-LC3-transfected LO2 cells (Fig. 9H,I), indicating the regulatory role of STVNa in autophagic flux.

## Discussion

NAFLD is a major cause of chronic kidney disease (CKD), which leads to severe liver-related complications and a high mortality rate^19^. Therefore, the development of an effective therapeutic strategy is of utmost urgency for treating patients with NAFLD. Our previous work showed that STVNa could improve lipid metabolism in CKD animal models^15^. In this study, we assessed the effect of STVNa on NAFLD rats and determined the involvement of SIRT1/AMPK-mediated autophagy during the progression of this disease. We observed that STVNa could exert a protective effect on NAFLD in HFD-fed rats. It has been reported that HFD-fed mice do not spontaneously develop from HS to NASH, and an additional lipid surplus is required^20–22^. During HS progression, HFD-fed mice display the main characteristics of NAFLD, including hyperlipidemia, IR and obesity^23,24^. STVNa was found to directly alleviate HS, prevent abnormal BW gain, reduce lipid levels and improves glucose tolerance, thus mediating the pathogenesis of NAFLD. HFD-induced NASH is associated with the dysregulated lipid efflux from hepatic cells through very-low density lipoprotein, which in turns leads to severe HS, inflammatory response and high transaminase activity within 4–6 weeks^25,26^. Consistently, the HFD-fed rats in our study had low BW, decreased TC and TG levels and increased hepatic lipid deposition. In addition, STVNa effectively reversed the high levels of AST and ALT, inflammatory cell infiltration and cell death HFD-fed rats, exhibiting the protective effect of STVNa on hepatic oxidative stress triggered by excess lipid accumulation. Moreover, STVNa exhibited lipid-lowering effect and reduced lipid deposition in FFA-exposed LO2 cells in a concentration-dependent fashion. Overall, both in vitro and in vivo data indicated the beneficial effect of STVNa on NAFLD rats.

Autophagy can maintain lipid homeostasis through the hydrolysis of intracellular lipid droplets^27,28^. A portion of lipid droplets is packed into the autophagosomea and degraded into FFA within the autolysosomea, and subsequently undergoes mitochondrial oxidation^28^. Previous research has shown that impaired autophagy is closely related to NAFLD development. Knockout of ATG5 (a molecular regulator of autophagosome elongation and formation) and treatment with 3-methyladenine (an inhibitor of autophagic activation) can lead to reduced lipid degradation, FFA β-oxidation, and ultimately excess lipid accumulation in FFA-exposed hepatic cells^29,30^. Few animal studies also demonstrated that nutrient depletion could initiate autophagy, promote LC3 lipidation, and increase the number of lipid droplets-containing autophagosomes^31,32^. These findings imply that autophagy can regulate the degradation of TG-containing lipid droplets in mice. Therefore, it can be inferred that STVNa has a strong therapeutic effect against NAFLD via induction of hepatic autophagy.

In this study, excess lipid deposition could impair hepatic autophagy in NAFLD rats. Notably, the levels of p62 (a selective substrate for autophagy) and LC3B-II (a specific biomarker for autophagosome production) were increased and decreased, respectively, in the liver of HFD-fed rats, suggesting the impairment of autophagy during NAFLD development. After treatment with STVNa, the decrease in p62 and increase in LC3B-II were observed, indicating that this agent could restore impaired autophagy in the liver of NAFLD rats. A growing body of evidence suggests that STVNa-mediated autophagic flux is associated with the decreased TG levels in FFA-exposed LO2 cells. STVNa is shown to improve autophagic flux, attenuate lipid deposition, and restore impaired autophagy in liver cells. Thus, it is speculated that STVNa can alleviate NAFLD by reinforcing autophagy and promoting lipid degradation.

AMPK is an essential regulator of cellular energy status, and can maintain energy homeostasis via autophagy^33^. The phosphorylation of AMPK is caused by increasing AMP:ATP ratios and metabolic stress, which in turns upregulates SIRT1 expression and ultimately induces autophagy^34,35^. In this study, STVNa reversed AMPK phosphorylation in NAFLD mice and elevated p-AMPK:AMPK ratios in NAFLD cells in a concentration-dependent fashion, demonstrating that STVNa could alleviate NAFLD by mediating autophagy via AMPK-dependent pathway. SIRT1 is a nicotinamide adenine dinucleotide (NAD+)-dependent histone deacetylase that regulates various cellular processes, and has been recognized as an important regulator of lipid metabolism via autophagy^36^. Our results showed that STVNa could reverse the downregulated expression of SIRT1 in HFD-fed rats, suggesting that the protective effect of STVNa on autophagy in NAFLD rats is primarily mediated bySirt1/AMPK pathway.

**Figure 10:**
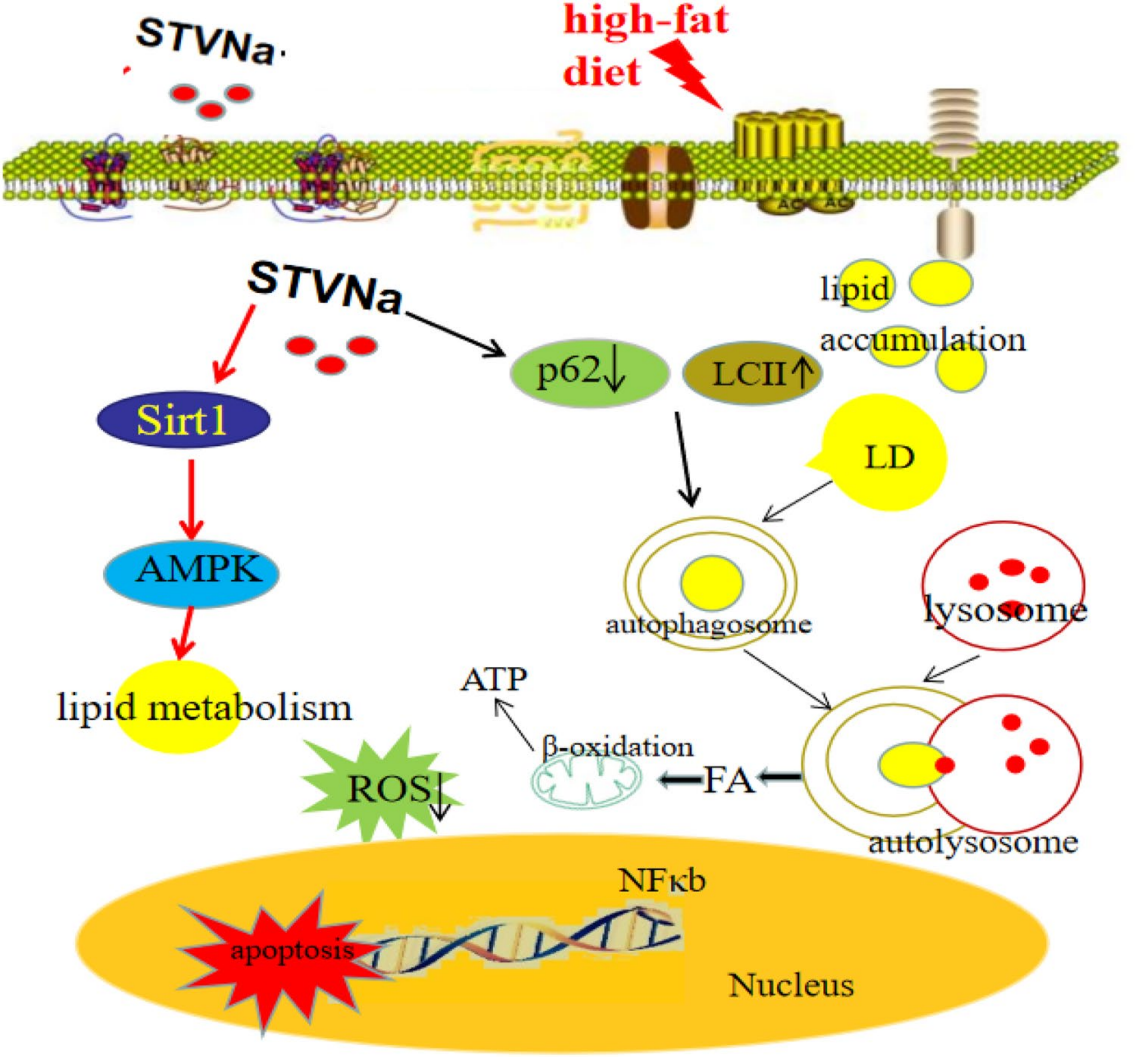
Mechanistic illustration of the effect of STVNa on FFA-induced NAFLD. STVNa increased SIRT1 expression, thus promoting lipid metabolism and inhibiting cellular ROS overload. In addition, STVNa reduced P62 expression, thereby inducing autophagy activation. Small lipid droplets are engulfed by LC3-II positive membranes, indicating that autophagy can degrade lipid droplets. Lipid cargoes are delivered to lysosomes for degradation by acid hydrolases. FFAs are transported into the cytosol and undergo mitochondrial β-oxidation for energy supply. These two mechanisms work together to reduce fat, thus improving NAFLD.

In summary, STVNa could attenuate lipid deposition in NAFLD rats by initiating autophagy via Sirt1/AMPK pathway (Fig. 10). Our findings provide a novel mechanism for the anti-NAFLD effect of STVNa, by which autophagy may play a vital role. Therefore, STVNa can serve as an alternative therapeutic agent for treatment of NAFLD.

## Supplementary Information

Supplementary Figures.

## Data Availability

The data and materials supporting this research are available from the authors upon request. If you wants to request the data from this study, please contacted YingMei, the first author.
