# Supplementary material for: Therapeutic effects of isosteviol sodium on non-alcoholic fatty liver disease by regulating autophagy via Sirt1/AMPK pathway
Source: Sci Rep. 2022 Jul 27;12:12857. doi: 10.1038/s41598-022-16119-0 (PMC9329321; doi:10.1038/s41598-022-16119-0)
Supplement: Supplementary file 1 — Supplementary Figures. [file 41598_2022_16119_MOESM1_ESM.pdf]

Therapeutic Effects of Isosteviol Sodium on Non-Alcoholic Fatty Liver Disease by Regulating Autophagy via Sirt1/AMPK pathway

Ying MEI[1,2], Hui HU[3], Liangjun Deng[3], Xiaoou SUN[*3], Wen TAN[*4]

1 School of Pharmacy, Jinan University, Guangzhou 510632, China
2 YZ Health-tech Inc., Hengqin District, Zhuhai 519000, China
3 Institute of Biomedical and Pharmaceutical Sciences, Guangdong University of Technology, Guangzhou 510006, China
4 Jeffrey Cheah School of Medicine and Health Sciences, Monash University Malaysia, Bandar Sunway 47500, Malaysia

* Corresponding author: Xiaoou Sun, Ph.D. Wen Tan, MD., Ph.D.
Xiaoou Sun, Institute of Biomedical and Pharmaceutical sciences, Guangdong University of Technology, Guangzhou 510006, China. xiaoousun@gdut.edu.cn, Tel: 13539850005

Wen Tan, Jeffrey Cheah School of Medicine and Health Sciences, Monash University Malaysia, Bandar Sunway 47500, Malaysia . went@gdut.edu.cn, Tel: 13928951505

Supporting Information 1. Western blotting results.
1) This file contains the original blot images of all the samples.
2) Guarantee 3 independent repeated experiments for every case.
3) The framed areas were used in the main text.

Figure4G

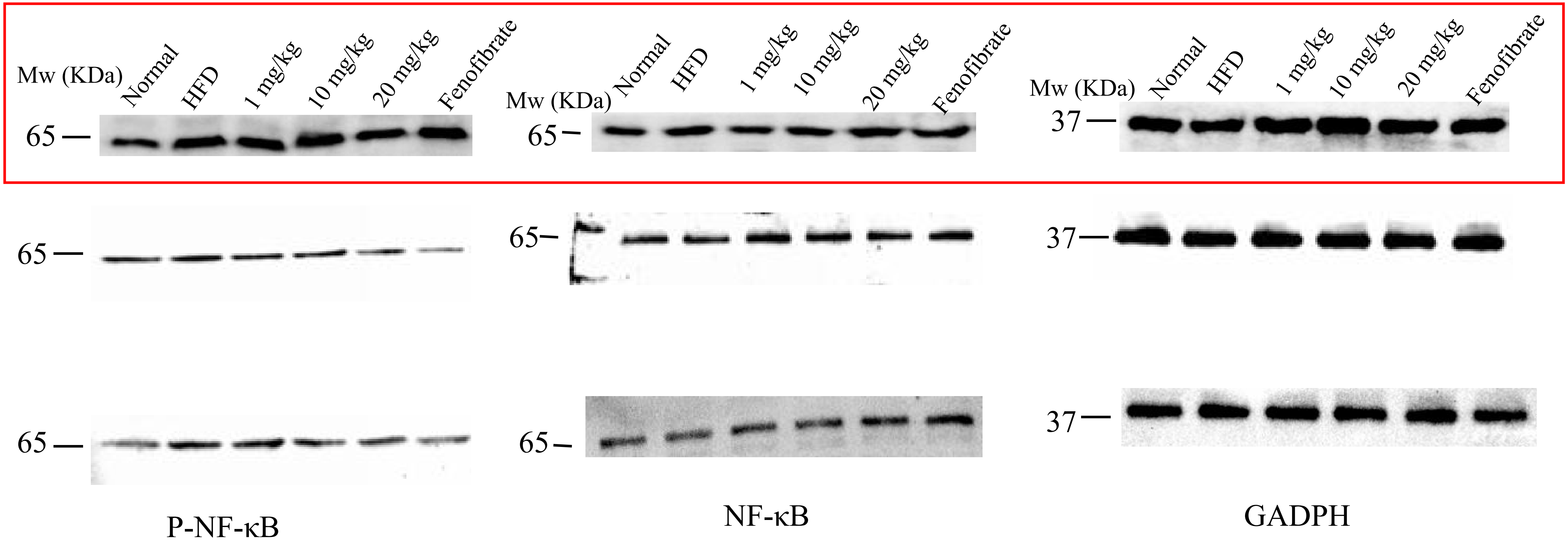
Mw (KDa)
Normal
HFD
1 mg/kg
10 mg/kg
20 mg/kg
Fenofibrate
65
37
P-NF-κB
NF-κB
GADPH

Figure7A

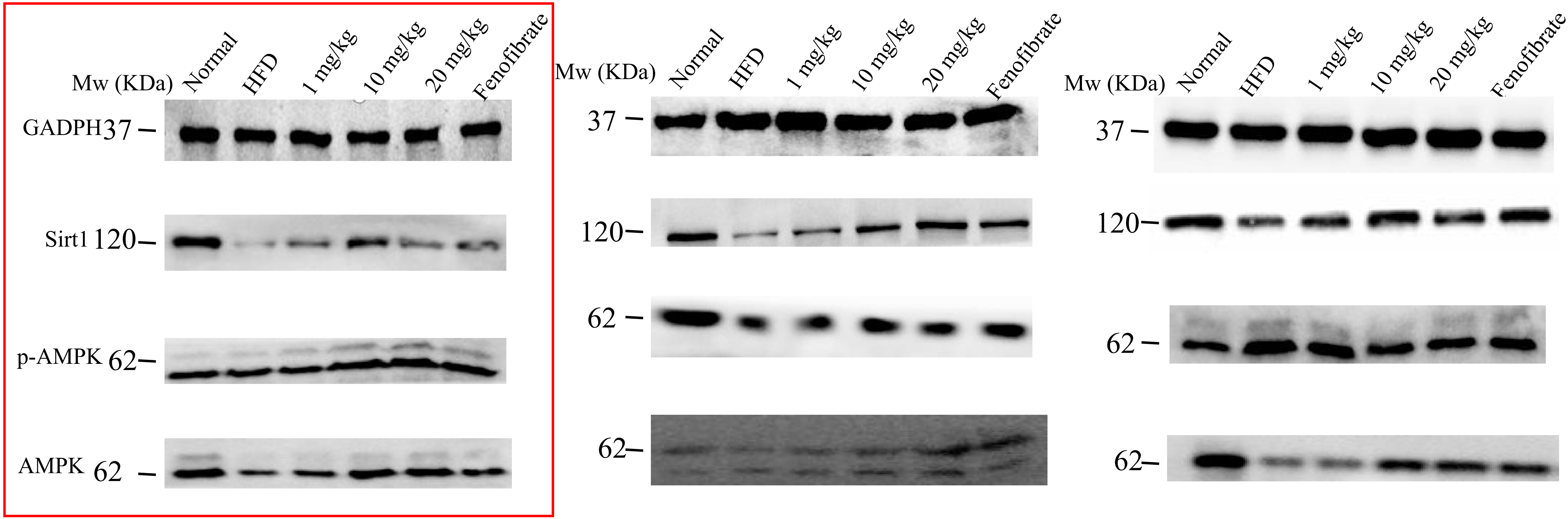
Mw (KDa)
Normal
HFD
1 mg/kg
10 mg/kg
20 mg/kg
Fenofibrate
GADPH
37
Sirt1
120
p-AMPK
62
AMPK
62
Mw (KDa)
Normal
HFD
1 mg/kg
10 mg/kg
20 mg/kg
Fenofibrate
37
120
62
62
Mw (KDa)
Normal
HFD
1 mg/kg
10 mg/kg
20 mg/kg
Fenofibrate
37
120
62
62

Figure7D

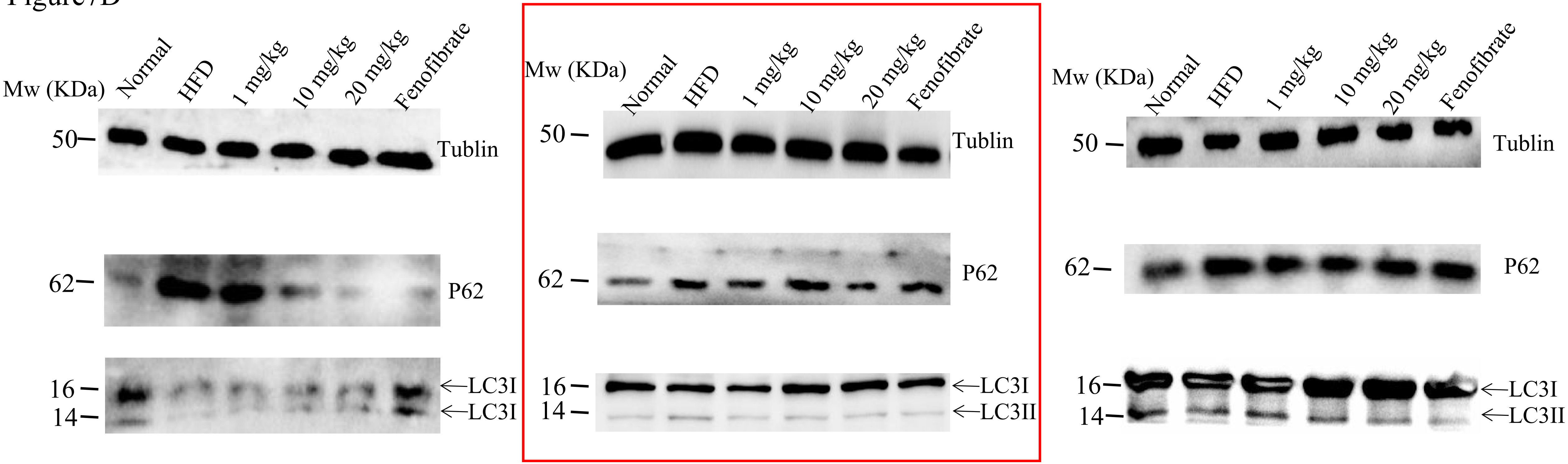
Mw (KDa)
Normal
HFD
1 mg/kg
10 mg/kg
20 mg/kg
Fenofibrate
50
Tublin
62
P62
16
14
←LC3I
←LC3I
Mw (KDa)
Normal
HFD
1 mg/kg
10 mg/kg
20 mg/kg
Fenofibrate
50
Tublin
62
P62
16
14
←LC3I
←LC3II
Mw (KDa)
Normal
HFD
1 mg/kg
10 mg/kg
20 mg/kg
Fenofibrate
50
Tublin
62
P62
16
14
←LC3I
←LC3II

Figure9A

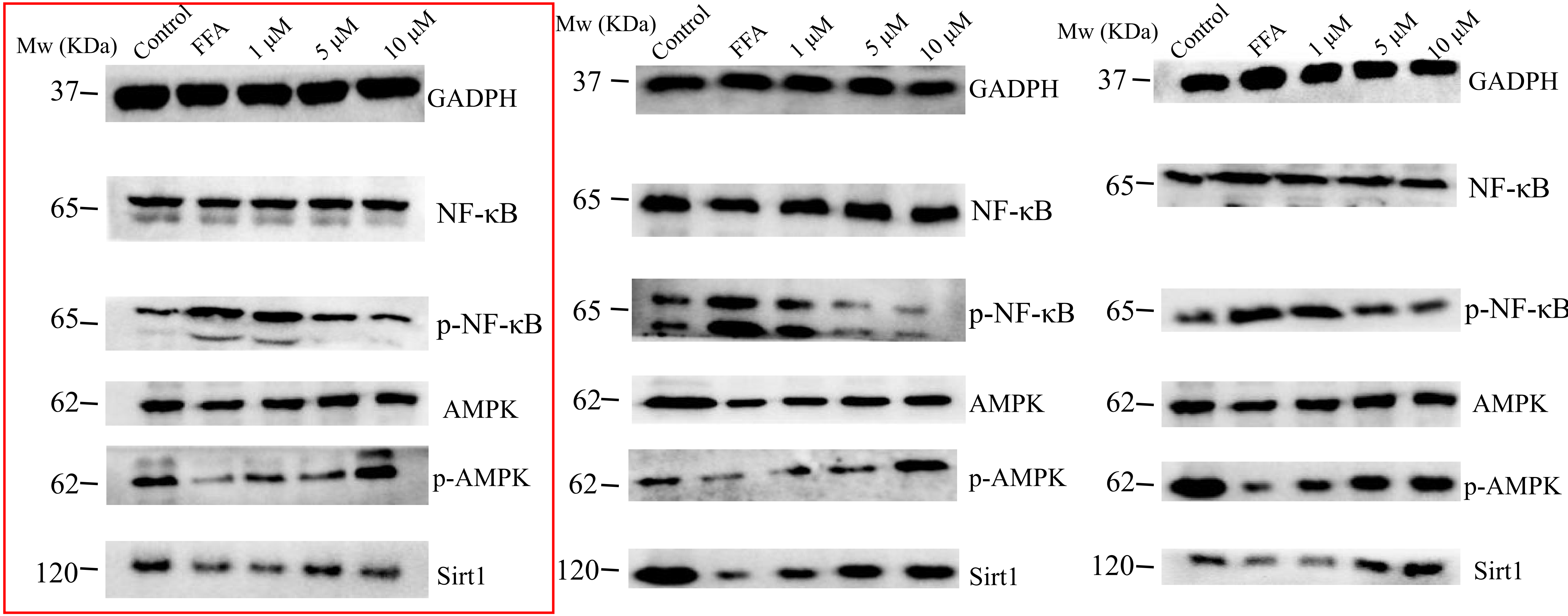

Mw (KDa)
Control
FFA
1 μM
5 μM
10 μM
37
GADPH
65
NF-κB
65
p-NF-κB
62
AMPK
62
p-AMPK
120
Sirt1
Mw (KDa)
Control
FFA
1 μM
5 μM
10 μM
37
GADPH
65
NF-κB
65
p-NF-κB
62
AMPK
62
p-AMPK
120
Sirt1
Mw (KDa)
Control
FFA
1 μM
5 μM
10 μM
37
GADPH
65
NF-κB
65
p-NF-κB
62
AMPK
62
p-AMPK
120
Sirt1

Figure9D

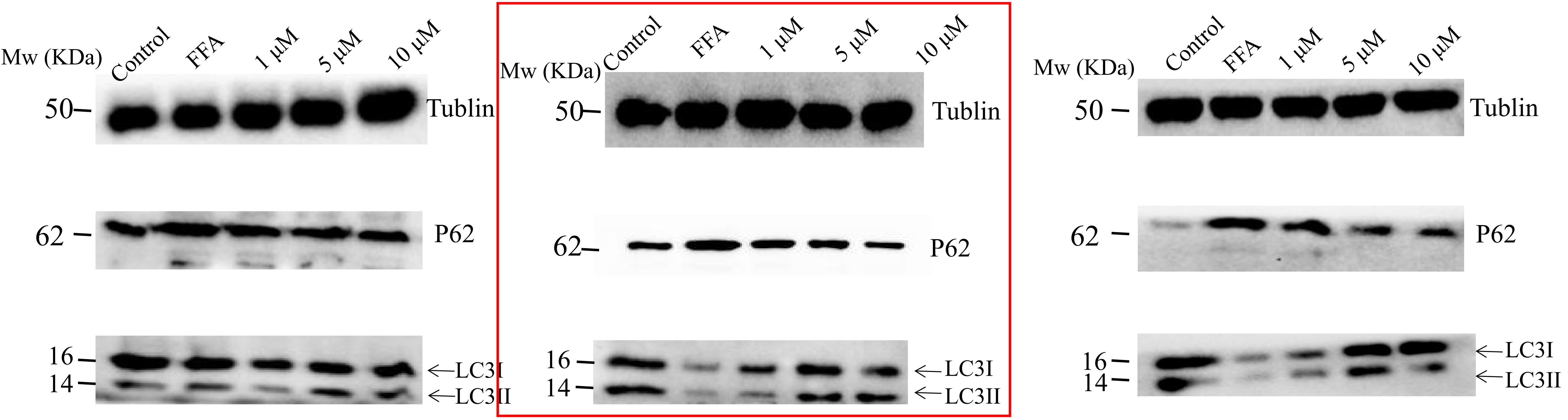

Mw (KDa)
Control
FFA
1 μM
5 μM
10 μM
50
Tublin
62
P62
16
14
LC3I
LC3II
Mw (KDa)
Control
FFA
1 μM
5 μM
10 μM
50
Tublin
62
P62
16
14
LC3I
LC3II
Mw (KDa)
Control
FFA
1 μM
5 μM
10 μM
50
Tublin
62
P62
16
14
LC3I
LC3II
